# Which psychological, social and physical environmental characteristics predict changes in physical activity and sedentary behaviors during early retirement? A longitudinal study

**DOI:** 10.7717/peerj.3242

**Published:** 2017-05-11

**Authors:** Delfien Van Dyck, Greet Cardon, Ilse De Bourdeaudhuij

**Affiliations:** 1 Research Foundation Flanders, Brussels, Belgium; 2 Faculty of Medicine and Health Sciences, Department of Movement and Sports Sciences, Ghent University, Ghent, Belgium

**Keywords:** Exercise, Healthy aging, Older adults, Sitting, Ecological model, Active living

## Abstract

**Background:**

In the context of healthy ageing, it is necessary to identify opportunities to implement health interventions in order to develop an active lifestyle with sufficient physical activity and limited sedentary time in middle-aged and older adults. The transition to retirement is such an opportunity, as individuals tend to establish new routines at the start of retirement. Before health interventions can be developed, the psychological, social and physical environmental determinants of physical activity and sedentary behaviors during early retirement should be identified, ideally with longitudinal studies. The aim of this paper was first to examine whether psychological, social and physical environmental factors at the start of retirement predict longitudinal changes in physical activity and sedentary behaviors during the first years of retirement. Second, moderating effects of gender and educational levels were examined.

**Methods:**

This longitudinal study was conducted in Flanders, Belgium. In total, 180 recently retired (>1 month, <2 years at baseline) adults completed a postal questionnaire twice (in 2012–2013 and two years later in 2014–2015). The validated questionnaire assessed socio-demographic information, physical activity, sedentary behaviors, and psychological, social and physical environmental characteristics. Multiple moderated hierarchic regression analyses were conducted in SPSS 22.0.

**Results:**

Higher perceived residential density (*p* < 0.001) and lower aesthetics (*p* = 0.08) predicted an increase in active transportation (adjusted *R*^2^ = 0.18). Higher baseline self-efficacy was associated with an increase in leisure-time physical activity (*p* = 0.001, adjusted *R*^2^ = 0.13). A more positive perception of old age (*p* = 0.04) and perceiving less street connectivity (*p* = 0.001) were associated with an increase in screen time (adjusted *R*^2^ = 0.06). Finally, higher baseline levels of modeling from friends (*p* = 0.06) and lower perceived land use mix access (*p* = 0.09) predicted an increase in car use (adjusted *R*^2^ = 0.06). A few moderating effects, mainly of educational level, were found.

**Discussion:**

Walkability characteristics (perceived residential density) and self-efficacy at the start of retirement are the most important predictors of longitudinal changes in active transportation and leisure-time physical activity. Few moderating effects were found, so health interventions at the start of retirement focusing on self-efficacy and specific walkability characteristics could be effective to increase physical activity in recently retired adults. No firm conclusions can be drawn on the importance of the examined predictors to explain change in car use and screen time, possibly other factors like the home environment, or automatic processes and habit strength are more important to explain sedentary behaviors.

## Introduction

Globally, life expectancy has increased steadily over the last decades. Between 2000 and 2050, the proportion of adults older than 60 years of age is expected to double from 11% to 22% (i.e., from about 605 million to more than a billion) (World Health Organization, 2014). This trend induces major societal challenges, like an increase in health care costs due to age-related chronic diseases (e.g., cardio-vascular diseases, type 2 diabetes, sarcopenia) (Organization for Economic Cooperation and Development, 2006). In middle and older age adopting and adhering to a healthy lifestyle with sufficient levels of physical activity (PA), limited sedentary time and a healthy diet, is needed to reduce risks for chronic diseases and mortality (Knoops et al., 2004; King, Mainous & Geesey, 2007). In that context, it is necessary to identify opportunities to develop healthy lifestyles in middle-aged and older adults in order to promote healthy ageing.

The transition to retirement can be seen as such an opportunity. Retirement can be defined as ‘a permanent and complete withdrawal from the labor force’ and goes together with important changes in time availability and flexibility, social networks, income and financial security, which can all impact adults’ lifestyles, both positively and negatively (Kim & Moen, 2002; Barnett, Van Sluijs & Ogilvie, 2012). Currently available evidence shows that PA and sedentary behaviors rather develop adversely during early retirement: total PA tends to decrease when making the transition to retirement, and this decrease is probably caused by a decrease in work- and transport-related PA that is insufficiently compensated by an increase in leisure-time PA (Slingerl et al., 2007; Touvier et al., 2010; Lahti et al., 2011; Barnett et al., 2014). Retirement has also been associated with an increase in specific sedentary behaviors like TV viewing and reading, and a decrease in occupational sitting and car use (Touvier et al., 2010; Barnett et al., 2014; Sprod et al., 2015; Van Dyck et al., in press).

Nonetheless, retirement can also be seen as a transition during which individuals rethink habitual behaviors and establish new routines (Jonsson, Josephsson & Kielhofner, 2001). Individuals who are about to retire or retired recently seem to be particularly receptive to behavior change (e.g., smoking cessation) (Lang et al., 2007). Therefore, early retirement seems to be a promising stage to implement interventions to stimulate middle-aged and older adults to develop/maintain a healthy lifestyle.

Before one can develop interventions aiming to increase PA and/or decrease sedentary behaviors during early retirement, it is necessary to identify the specific psychological, social and physical environmental determinants of these behaviors during early retirement. In health research, socio-ecological frameworks are often used the examine the multi-dimensional determinants of PA (Sallis, Owen & Fisher, 2008). The socio-ecological framework for PA has been adapted to examine the potential determinants of sedentary behaviors, but empirical evidence on these determinants is lacking, especially in older adults (Owen et al., 2011). A few qualitative studies examined these determinants during early retirement, mainly by conducting focus group interviews with the target group (Barnett, Guell & Ogilvie, 2012; McDonald et al., 2015; Kosteli, Williams & Cumming, 2016; Van Dyck et al., in press). These studies identified that several intrapersonal (e.g., self-efficacy, self-regulatory strategies, outcome expectations, social norms and beliefs on ageing and retirement, need for personal challenges, perceived health benefits of PA, financial constraints, loss of daily structure), interpersonal (e.g., social support, social roles and responsibilities) and physical environmental factors (e.g., opportunities to be active, physical barriers like poorly maintained sidewalks) can be important factors for (insufficient) PA in recently retired adults. To our knowledge, only one qualitative study already examined potential determinants of sedentary behaviors during early retirement (Van Dyck et al., in press) and concluded that in this age group knowledge on the negative effects of sedentary behaviors is absent, inducing a lack of motivation to decrease sedentary behaviors.

In addition to the qualitative evidence, quantitative studies are also needed to confirm the importance of the determinants that emerged from previous focus group studies. Although qualitative studies are very useful, they usually include small study samples. Consequently, quantitative studies with larger samples are more appropriate to draw conclusions for ‘the overall population’. To our knowledge, no previous quantitative studies examined the multidimensional correlates of PA and sedentary behaviors in this specific target group of recently retired adults. Ideally, such studies should use a longitudinal design in order to draw conclusions on whether specific factors at the start of retirement predict changes in PA and sedentary behaviors during early retirement, and to make it possible to develop effective health interventions focusing on specific determinants at the start of retirement.

Therefore, the first aim of this paper was to examine whether psychological, social and physical environmental factors at the start of retirement predict longitudinal changes in leisure-time PA, active transportation, screen time and car use during the first years of retirement. Because changes in PA and sedentary behaviors during early retirement have been shown to be dependent of gender and variations in socio-economic status (Mein et al., 2005; Chung et al., 2009; Barnett, Van Sluijs & Ogilvie, 2012; Barnett et al., 2014), the second aim was to examine whether gender and educational level moderate the associations examined in the first aim.

## Materials and Methods

This study was conducted in Ghent (250,000 inhabitants, 156.18 square km (60.3 square miles), 1,601 inhabitants/square km), Flanders, Belgium. Baseline data were collected in two waves, a first wave in December 2012 and a second wave in May 2013. Follow-up data were similarly collected in two waves, two years after baseline data collection (December 2014, May 2015).

### Procedures and participants

The data used for this paper were part of a larger study in adults around retirement age where at baseline, individuals who retired recently (>1 month, <5 years of retirement) and individuals who planned to retire within the next 18 months were targeted. However, because this paper aims to examine whether psychological, social and physical environmental factors assessed at the beginning of retirement, can predict changes in PA and sedentary behaviors during early retirement, only the data of individuals who are at the start of their retirement (>1 month, <2 years of retirement) are used. This definition of ‘early retirement’ was chosen after consulting sessions with experts in health research in older adults and after four focus group sessions with retired adults. These focus groups were conducted in the scope of another study in the same target group (Van Dyck et al., in press). More details on the procedures of the larger study and the selection of the analytical sample for the current paper, can be found below.

In Flanders, the formal retirement age of the current workforce over 50 years of age lies between 58 and 65 years (Federal Pensions Service Belgium, 2016), but official records with information on retirement status are not publicly available. Consequently, the Public Service of Ghent selected a random sample of 7,500 58–65 year old adults from the municipal register for the study. At baseline, all these adults received an invitation letter with information on the study (2,500 adults in December 2012, 5,000 adults in May 2013). Only adults who planned to retire within the next 18 months, and those who had been retired for more than one month but less than five years could participate in the large-scale study. Retired adults needed to be fully retired from their main occupation, but engaging in voluntary work was allowed. Furthermore, as PA was one of the outcome variables, participants had to be able to walk 100 m without assistance in order to be eligible. Adults who were willing to participate in the study and met the inclusion criteria, received a postal questionnaire (with a pre-stamped envelope to return the questionnaire) including questions on socio-demographic characteristics, psychological, social and physical environmental factors, PA and sedentary behaviors, and physical and mental health. In total, 597 adults (455 retired, 142 planning to retire) returned a complete questionnaire. Because it is unknown how many of the 7,500 addressed adults were eligible to participate in the study, it is not possible to calculate the response rate.

After two years (December 2014 and May 2015) these 597 adults received the same postal questionnaire again (follow-up measurements). In total, 463 adults (77.6%) returned a complete questionnaire at follow-up. Of these 463 participants, five were not yet retired, three did not report the month/year of retirement, and 9 participants had not been working before they officially retired (seven housewives and two disabled persons). Consequently, the final sample that completed both baseline and follow-up measurements of the large-scale study consisted of 446 participants (341 adults who were already retired at baseline and 105 adults who retired between baseline and follow-up). For this paper, the 105 adults who were not retired yet at baseline and adults who had been retired for more than two years at baseline (*n* = 161) were excluded from the analyses. This led to a final analytical sample of 180 adults who were at the start of retirement at baseline.

The study protocol was approved by the ethics committee of the Ghent University Hospital (B670201215326). Written informed consent was obtained from all participants.

### Measures

#### Dependent variables: changes in physical activities and sedentary behaviors

Self-reported PA was assessed with the International Physical Activity Questionnaire (IPAQ; long past seven days version; available at http://www.ipaq.ki.se.) PA assessed by the IPAQ showed good reliability (intra-class correlations range from 0.46 to 0.96) and fair-to -moderate criterion validity compared against accelerometers (median *ρ* = 0.30) in a 12-country study (Craig et al., 2003). Frequency (number of days) and duration (minutes/day) of PA in different domains were queried. Based on this information, separate estimates of weekly minutes of active transportation (sum of walking and cycling for transport) and leisure-time PA (sum of leisure-time walking, cycling and moderate-to-vigorous PA (MVPA)) were calculated.

Self-reported minutes/week of car use and screen time (sum of TV viewing time and computer use) were assessed using a translated (Flemish) version of the leisure-time sedentary behavior questionnaire developed by Salmon and collegues (2003). The English-language version of the questionnaire has fair to excellent reliability (intra-class range from 0.56 to 0.82). Concurrent validity, assessed against a three-day behavioral log was fair-to-moderate, with rho’s ranging from 0.20 to 0.60 (Salmon et al., 2003).

#### Predictors: psychological, social and physical environmental characteristics

All psychological variables assessed in the questionnaire were derived from previous studies in adults, older adults and adolescents (Marcus et al., 1992; De Bourdeaudhuij & Sallis, 2002; Deforche et al., 2004; De Bourdeaudhuijet al., 2005; Van Holle et al., 2015; English Longitudinal Study of Ageing, available at http://elsa-project.ac.uk). Five categories of psychological variables were included: perceived benefits of PA, perceived barriers towards PA, self-efficacy, perceptions of retirement and perceptions of old age. Scales were constructed for perceived benefits of PA (e.g., losing weight, enjoyment; mean of six items, Cronbach’s alpha (*α*) = 0.56), perceived barriers (e.g., feeling to old, fear for injuries, bad weather; mean of 11 items, *α* = 0.87), self-efficacy (e.g., being active even when not feeling well, being active even without a sport partner; mean of five items, *α* = 0.82) and perceptions of old age (e.g., old age is accompanied by loneliness, we can learn a lot from old people; mean of 11 items, *α* = 0.65). Perceptions of retirement consisted of two items that were analyzed separately: ‘I perceive retirement as a start for slowing down’ and ‘I perceive retirement as a start for a more active lifestyle’. All items were scored on a five-point scale with a higher score reflecting more positive psychological profiles, except for the self-efficacy items, which were scored on a three-point scale (I know I cannot, I think I can, I know I can).

Social variables included modelling, social support and social cohesion of the neighborhood. All variables were derived from previous studies in adults (Sallis et al., 1987; Sampson, Raudenbush & Earls, 1997; De Bourdeaudhuij & Sallis, 2002). Modelling consisted of three items (modelling from partner, friends, (grand)children) that were assessed using a seven-point scale (higher score = more perceived modelling). These items were analyzed separately due to low internal consistency (*α* < 0.50). Scales were constructed for social support (e.g., how often do friends support you to be active; six items, *α* = 0.85) and social cohesion of the neighborhood (e.g., people in my neighborhood can be trusted, this is a close-knit neighborhood; five items, *α* = 0.82). These items were scored on a five-point scale ranging from strongly disagree to strongly agree (higher score = more positive social characteristics).

To assess perceived physical environmental factors, the Dutch version of the NEWS questionnaire was used (De Bourdeaudhuij, Sallis & Saelens, 2003). Physical environmental subscales included were residential density, land use mix diversity, land use mix access, street network connectivity, infrastructure and safety for walking and cycling, traffic safety, crime safety and aesthetics. Land use mix diversity can be defined as ‘the level of integration of different types of uses for physical space within an area, including residential, office, retail/commercial, institutional, industry and public space’ (Saelens, Sallis & Frank, 2003). Land use mix access refers to the accessibility (e.g., distance and presence/absence of physical barriers) of destinations. Street network connectivity can be defined as ‘the directness or ease to travel between two points that is directly related to the characteristics of street design’ (e.g., many intersections, few dead-end-streets) (Saelens, Sallis & Frank, 2003). Calculation of these subscales was based on the official NEWS scoring guidelines (available at http://sallis.ucsd.edu), with a higher score reflecting a more positive environmental perception. The Dutch NEWS has acceptable to good reliability (intraclass correlation coefficients between 0.40 and 0.97) and acceptable validity (coefficients between 0.21 and 0.91) (De Bourdeaudhuij, Sallis & Saelens, 2003). All environmental factors were rated on a four-point scale, except for residential density (three-point scale) and land use mix diversity (five-point scale).

#### Socio-demographic covariates and moderators

Self-reported socio-demographics included gender, age, weight, height and educational level (primary, secondary, tertiary education). BMI was calculated by dividing the weight (kg) by the height (m) squared. For the analyses, educational level was dichotomized into high education (i.e., tertiary education) versus low education (i.e., primary and secondary education).

### Statistical analyses

All data were analyzed using SPSS 22.0 and multiple moderated hierarchic regression analyses were conducted to answer the research questions. In a first step, measures of change in PA (active transportation and leisure-time PA) and sedentary behaviors (screen time and car use) between baseline and follow-up were created by regressing the PA and sedentary behavior measures at follow-up onto their respective baseline values. Based on these regression outcomes, residualized change scores were computed. These scores can be interpreted as the amount of increase/decrease in PA or sedentary time between baseline and follow-up, independent of baseline scores. Furthermore, they eliminate autocorrelated error and regression to the mean effects and are therefore preferable to simple change scores (Cohen & Cohen, 1985; Bland & Altman, 1994).

In a second step, multicollinearity (*r* > 0.60) between the predictors (psychological, social and physical environmental characteristics) was analyzed. Only between perceived land use mix access and land use mix diversity (*r* = 0.65) and between self-efficacy and perceived barriers towards PA (*r* = 0.62) multicollinearity was present. Consequently, only the predictor with the strongest correlation with the dependent variable was included in the regression analyses. In a third step, bivariate correlations between the potential predictors and the outcome variables were examined. Only predictors that had a correlation with the dependent variable of *p* < 0.15 were included in the regression models (Tabachnik & Fidell, 2007).

In a final step, multiple moderated hierarchic regression analyses were conducted to examine whether psychological, social and physical environmental factors at the start of retirement predicted changes in PA and sedentary behaviors during early retirement, and the moderating effects of gender and educational level. Eight regression models were constructed, two for each dependent variable (residualized change scores of active transportation, leisure-time PA, screen time and car use). In a first block, the socio-demographic covariates (gender, age, BMI, educational level) were entered. In a second block, the psychological, social and physical environmental factors (predictors; baseline values) were entered as independent variables. In the third block, the cross-products (gender × predictor or educational level × predictor) were added to examine the moderating effects of gender (four models, one for each dependent variable) and educational level (four models). In case of significance, separate regression models (men versus women or high versus low educational level) were run to interpret the direction of the interaction. Statistical significance was set at 0.05 but because of the small study sample, marginally significant results (*p* < 0.10) were also reported. In physical activity research it is common to report on marginally significant findings when the sample size is limited (e.g.,Van Holle et al., 2016; De Cocker et al., 2016).

**Table 1:** Baseline descriptive characteristics of the study sample (*n* = 180).

Variable	
**Socio-demographic covariates**	
Gender (%)	
Men	48.6
Women	51.4
Age (mean (SD))	62.5 (2.1)
Educational level (%)	
High educational level	54.5
Low educational level	45.5
Body Mass index (mean (SD))	25.4 (3.9)
**Predictors** (mean (SD))	
*Psychological factors*	
Perceived benefits^a^	3.6 (0.6)
Perceived barriers^a^	4.1 (0.7)
Self-efficacy^b^	2.1 (0.54)
Perceptions of retirement^a^	
Retirement = slowing down	2.5 (1.1)
Retirement = more active life	3.2 (1.1)
Perception of old age^a^	3.5 (0.5)
*Social factors*	
Modelling from partner^c^	4.5 (2.2)
Modelling from friends^c^	4.2 (1.9)
Modelling from (grand)children^c^	5.0 (1.5)
Social support^a^	3.6 (1.0)
Neighborhood social cohesion^a^	3.6 (0.7)
*Physical environmental factors*	
Residential density^b^	192.6 (77.8)
Land use mix diversity^a^	3.0 (0.8)
Land use mix access^d^	3.2 (0.8)
Street network connectivity^d^	2.9 (0.5)
Infrastructure and safety for	2.5 (0.5)
Walking and cycling^d^	
Traffic safety^d^	2.5 (0.6)
Crime safety^d^	3.0 (0.6)
Aesthetics^d^	2.5 (0.6)
**Dependent variables** (mean (SD))	
*Physical activity (min/week)*	
Active transportation	240.8 (253.0)
Leisure-time physical activity	312.4 (359.2)
*Sedentary behaviors (min/week)*	
Screen time	1425.4 (777.2)
Car use	320.0 (387.8)

**Notes.**

a Positively scored on a five-point scale.

b Positively scored on a three-point scale.

c Positively scored on a seven-point scale.

d Positively scored on a four-point scale.

## Results

Baseline descriptive statistics of the study sample are presented in Table 1. In summary, 48.6% of the sample was male, mean age was 62.5 (2.1) years, 45.5% of the sample had a low educational level (i.e., no college or university degree) and mean BMI was 25.4 (3.9) kg/m^2^. Average values of the psychological, social and physical environmental factors, and of the outcome variables (PA and sedentary behaviors) can also be found in Table 1.

For change in active transportation, the following nine predictors were included in the regression model: self-efficacy, perceiving retirement as a start for slowing down, modelling from (grand)children, neighborhood social cohesion, residential density, land use mix access, street connectivity, infrastructure/safety for walking and cycling and aesthetics. Six predictors were included in the model for change in leisure-time PA: perceived benefits, self-efficacy, perceiving retirement as a start for being active, modelling from partner, land use mix access and traffic safety. Perception of old age and street connectivity were included as predictors in the model for change in screen time. Finally, modelling from friends, residential density and land use mix access were included in the model for change in car use.

### Psychological, social and physical environmental predictors of changes in PA and moderating effects of gender and educational level

Results of the regression analyses are presented in Table 2. Perceived residential density (*β* = 0.41, *p* < 0.001) and perceived aesthetics (*β* =  − 0.18, *p* = 0.08) were (marginally) significant predictors of changes in active transportation (adjusted *R*^2^ = 0.179). Higher perceived residential density and lower perceived aesthetics at baseline were related to an increase in active transportation. Educational level moderated the relation between modelling from (grand)children and change in active transportation (*β* = 0.66, *p* = 0.09). In participants with a low educational level, no association was found between modelling from (grand) children and change in active transportation (*β* = 0.06, *p* = 0.68) whereas in participants with a high educational level, higher modelling from (grand)children was related to an increase in active transportation (*β* = 0.22, *p* = 0.06). No other moderating effects, neither of educational level, nor of gender could be identified with regard to change in active transportation.

Regarding change in leisure-time PA, baseline self-efficacy was the only significant predictor (*β* = 0.32, *p* = 0.001; adjusted *R*^2^ = 0.134). Higher baseline levels of self-efficacy were associated with an increase in leisure-time PA. Educational level moderated the relation between perception of retirement (i.e., retirement is a start for a more active lifestyle) and change in leisure-time PA (*β* =  − 0.67, *p* = 0.045). In participants with a low educational level, a more positive perception of retirement was related to an increase in leisure-time PA (*β* = 0.35, *p* = 0.002) while in participants with a high educational level, no association was found (*β* = 0.06, *p* = 0.56). No other moderating effects of educational level or gender could be identified.

### Psychological, social and physical environmental predictors of changes in sedentary behaviors and moderating effects of gender and educational level

Results of the regression analyses are presented in Table 3. Baseline perception of old age (*β* = 0.16, *p* = 0.04) and perceived street connectivity (*β* =  − 0.25, *p* = 0.001) were significantly related to change in screen time (adjusted *R*^2^ = 0.064). A more positive perception of old age and perceiving less street connectivity were associated with an increase in screen time. Educational level and gender were not significant moderators of any of the associations.

**Table 2:** Multiple moderated hierarchic regression analyses: associations with changes in PA and moderating effects of educational level and gender.

Dependent variable	Predictors	Adj *R*^2^	*β* value	95% CI	*p*-value
Change in active transportation	Block 1 (sociodemographic covariates)	0.009			
	Block 2	0.188			
	Self-efficacy		0.01	−0.31, 0.37	0.88
	Retirement = slowing down		0.10	−0.08, 0.25	0.30
	Modelling from (grand)children		0.12	−0.04, 0.19	0.22
	Neighborhood social cohesion		−0.05	−0.35, 0.20	0.60
	**Residential density**		**0.41**	**0.002, 0.008**	**<0.001**
	Land use mix access		0.07	−0.18, 0.34	0.55
	Street network connectivity		−0.12	−0.64, 0.19	0.28
	Infrastructure/safety for walking and cycling		0.11	−0.27, 0.70	0.39
	**Aesthetics**		** − 0.18**	**−0.58, 0.03**	**0.08**
	Block 3A^a^	0.180			
	Educational level × self-efficacy		0.21	−0.53, 0.85	0.64
	× retirement = slowing down		−0.03	−0.38, 0.34	0.91
	**× modelling from (grand)children**		**0.66**	**−0.04, 0.50**	**0.09**
	× neighborhood social cohesion		0.39	−0.44, 0.81	0.56
	× residential density		0.02	−0.005, 0.006	0.94
	× land use mix access		0.76	−0.16, 1.03	0.15
	× street network connectivity		0.36	−0.69, 1.15	0.62
	× infrastructure/safety for walking and cycling		−0.22	−1.21, 0.90	0.77
	× aesthetics		−0.02	−0.77, 0.74	0.97
	Block 3B^b^	0.152			
	Gender × self-efficacy		−0.30	−0.96, 0.47	0.50
	× retirement = slowing down		0.07	−0.35, 0.43	0.84
	× modelling from (grand)children		0.37	−0.13, 0.38	0.33
	× neighborhood social cohesion		0.23	−0.46, 0.68	0.70
	× residential density		−0.47	−0.01, 0.002	0.22
	× land use mix access		0.31	−0.44, 0.77	0.59
	× street network connectivity		0.67	−0.45, 1.28	0.34
	× infrastructure/safety for walking and cycling		−0.72	−1.57, 0.54	0.34
	× aesthetics		−0.41	−0.96, 0.39	0.41
Change in leisure-time PA	Block 1 (sociodemographic covariates)	0.065			
	Block 2	0.199			
	Perceived benefits		0.03	−0.25, 0.35	0.74
	**Self-efficacy**		**0.32**	**0.26, 0.94**	**0.001**
	Retirement = more active life		0.08	−0.09, 0.24	0.37
	Modelling from partner		0.05	−0.05, 0.10	0.55
	Land use mix access		0.06	−0.14, 0.28	0.51
	Traffic safety		0.14	−0.04, 0.45	0.10
	Block 3A^a^	0.204			
	Educational level × perceived benefits		0.30	−0.48, 0.79	0.63
	× self-efficacy		0.26	−0.46, 0.88	0.54
	**× retirement = more active life**		**−0.68**	**−0.69, −0.01**	**0.045**
	× modelling from partner		−0.17	−0.21, 0.10	0.39
	× land use mix access		0.18	−0.34, 0.54	0.65
	× traffic safety		0.14	−0.04, 0.45	0.10
	Block 3B^b^	0.168			
	Gender × perceived benefits		0.35	−0.52, 0.89	0.61
	× self-efficacy		−0.17	−0.84, 0.55	0.68
	× retirement = more active life		−0.05	−0.38, 0.32	0.88
	× modelling from partner		0.07	−0.13, 0.18	0.76
	× land use mix access		−0.34	−0.64, 0.26	0.41
	× traffic safety		−0.26	−0.71, 0.34	0.49

**Notes.**

a Block 3A: regression model with educational level as a moderator.

b Block 3B: regression model with gender as a moderator.

PA physical activity CI confidence interval Adj adjusted

**Table 3:** Multiple moderated hierarchic regression analyses: associations with changes in sedentary behaviors and moderating effects of gender and educational level.

Dependent variable	Predictors	Adj *R*^2^	*β* value	95% CI	*p*-value
Change in screen time	Block 1 (sociodemographic covariates)	0.052			
	Block 2	0.116			
	**Perception of old age**		**0.16**	**0.02, 0.61**	**0.04**
	**Street network connectivity**		**−0.25**	**−0.69, −0.17**	**0.001**
	Block 3A^a^	0.105			
	Educational level × perception of old age		0.02	−0.59, 0.62	0.97
	× street network connectivity		−0.04	−0.54, 0.50	0.93
	Block 3B^b^	0.105			
	Gender × perception of old age		−0.07	−0.65, 0.57	0.91
	× street network connectivity		0.11	−0.46, 0.60	0.80
Change in car use	Block 1 (sociodemographic covariates)	−0.015			
	Block 2	0.058			
	**Modelling from friends**		**0.21**	**−0.01, 0.24**	**0.06**
	Residential density		−0.14	−0.005, 0.001	0.26
	**Land use mix access**		**−0.20**	**−0.54, 0.04**	**0.09**
	Block 3A^a^	0.084			
	**Educational level × modelling from friends**		**0.63**	**−0.01, 0.51**	**0.06**
	× Residential density		−0.20	−0.01, 0.004	0.54
	× Land use mix access		−0.67	−1.11, 0.27	0.23
	Block 3B^b^	0.080			
	**Gender × modelling from friends**		**−0.54**	**−0.47, 0.02**	**0.08**
	× Residential density		0.05	−0.006, 0.007	0.89
	× Land use mix access		0.57	−0.26, 0.95	0.26

**Notes.**

a Block 3A: regression model with educational level as a moderator.

b Block 3B: regression model with gender as a moderator.

PA physical activity CI confidence interval

Regarding change in car use, baseline modeling from friends (*β* = 0.21, *p* = 0.06) and perceived land use mix access (*β* =  − 0.20, *p* = 0.09) were (marginally) significant predictors (adjusted *R*^2^ = 0.058). Higher baseline levels of modeling from friends and lower perceived land use mix access were associated with an increase in car use. Educational level and gender were marginally significant moderators of the relation between modelling from friends and change in car use (*β* = 0.63, *p* = 0.06 and *β* =  − 0.54, *p* = 0.08). In females (*β* =  − 0.01, *p* = 0.94) and participants with a low educational level (*β* = 0.04, *p* = 0.83) no relation could be identified. In males (*β* = 0.23, *p* = 0.10) and participants with a high educational level (*β* = 0.24, *p* = 0.07), higher baseline levels of modelling from friends were marginally significantly associated with an increase in car use. No other moderating effects were found.

## Discussion

To our knowledge, this was the first quantitative study with a longitudinal design examining the multidimensional correlates of PA and sedentary behavior in recently retired adults. This paper aimed to examine whether psychological, social and physical environmental factors at the start of retirement can predict longitudinal changes in leisure-time PA, active transportation, screen time and car use during the first years of retirement. Furthermore, potential moderating effects of gender and educational level were examined.

Overall, the results showed that only a limited number of the included factors were associated with changes in PA and sedentary behaviors. Some moderating effects, mainly of educational level, were found, suggesting that to a certain extent, distinct approaches could be preferable to optimally reach high- and low-educated individuals in future interventions. However, as most of these moderating effects of educational level were only marginally significant, no definite conclusions can be drawn. Future studies with larger study samples are needed to examine this more thoroughly. Furthermore, the explained variance of the correlates was considerably larger for changes in PA (13.4% for change in leisure-time PA and 17.9% for change in active transportation) than for changes in sedentary behaviors (5.8% for change in car use and 6.4% for change in screen time). This could be due to the fact that the content of the questions to assess the psychological, social and physical environmental factors primarily focused on PA and not specifically on sedentary behaviors; for instance, modelling was assessed by asking how often the participants’ partner/friends/(grand) children were physically active, and perceived barriers/benefits were queried towards PA. Only the questions about participants’ perceptions of retirement, perceptions of old age, social cohesion of the neighborhood and specific physical environmental factors (e.g., residential density, aesthetics) were more general and did not focus specifically on PA. Until now, almost no studies examining potential correlates of sedentary behaviors used questions specifically related to sedentary behaviors, mainly because this type of research is still in its infancy, and little is known about its potential correlates. Also theoretical frameworks should be fine-tuned and tested empirically: the socio-ecological framework of PA (Sallis, Owen & Fisher, 2008) has been adapted for sedentary behavior (Owen et al., 2011) and is now being increasingly used in sedentary behavior research, but it seems that more thorough adaptation and the inclusion of novel sedentary-specific correlates is still needed. In that context, Chastin et al. (2016) recently introduced the Systems Of Sedentary behaviors (SOS) framework, an adaptation of the framework of Owen et al. (2011). The SOS framework has been developed based on literature review and expert meetings, and is a transdisciplinary model taking into account six clusters of potential determinants of sedentary behavior. Future research should empirically examine the theoretical correlates of the SOS framework in order to find out whether including these factors can increase the explained variance.

When examining the results in more detail, it can be concluded that the physical environmental perceptions (residential density) were important to explain changes in active transportation. Also in previous (cross-sectional) studies in adults and older adults, transportation PA was more frequently related to the physical environment than recreational PA (Van Cauwenberg et al., 2011; Van Holle et al., 2012). Walkability characteristics like residential density and land use mix access have been consistently associated with active (and passive) transportation in adults (Van Holle et al., 2012), but evidence in older adults is less consistent (Van Cauwenberg et al., 2011; Bauman et al., 2012). The present study adds evidence for the importance of the built environment in recently retired adults and confirms the assumption that living in a high walkable neighborhood can be beneficial to increase active transportation. Therefore, as emphasized in previous studies in adults (Heath et al., 2006; Bauman et al., 2012; Sallis et al., 2016), health interventions and policy makers should pay attention to optimizing the walkability of neighborhoods for active transportation, and to make people who live in less walkable environments aware of the possibilities that do exist in their neighborhood (e.g., recreational facilities). In that way, not only adults, but also the specific group of recently retired adults can potentially be reached.

Self-efficacy (i.e., confidence in the ability to be active without a sport partner, when not having a lot of time, when not feeling well and when the weather is not good) was the only factor that predicted a positive change in leisure-time PA in the total sample. Previous studies have shown that self-efficacy is amongst the most important determinants of leisure-time PA in (older) adults (Caudroit, Stephan & Le Scanff, 2011; Koeneman et al., 2011; Bauman et al., 2012; Van Stralen et al., 2009). Nonetheless, it was somewhat surprising that no other psychological or social factors were related to change in leisure-time PA since results of previous qualitative studies in recently retired adults identified several specific intra- and interpersonal correlates of leisure-time PA, like social support, perceived health benefits and financial constraints (Barnett, Guell & Ogilvie, 2012; McDonald et al., 2015; Kosteli, Williams & Cumming, 2016; Van Dyck et al., in press). These variables were also assessed in our study, but were not related to the outcome measures. The current quantitative study could not confirm these previous qualitative results. The importance of self-efficacy confirms that future interventions should be multi-dimensional and combine an individual with an environmental focus, in order to increase different types of PA in recently retired adults (Sallis, Owen & Fisher, 2008).

Unexpected results were found regarding the correlates of change in screen time: adults with positive perceptions of old age and those who perceived higher street connectivity showed an increase in screen time. These findings are opposite to what one would expect from the socio-ecological framework for sedentary behavior (Owen et al., 2011). In this framework it is suggested that perceiving high street connectivity and having a positive psychosocial profile would be related to less sedentary time. This may confirm the assumption that correlates that focus on PA seem not really suitable to be linked to sedentary behaviors. Previous literature on the multidimensional correlates of sedentary behaviors in (older) adults is very limited, but the available evidence also showed inconsistent associations of street connectivity and other aspects of the physical environment with sedentary behaviors: some studies found negative associations (Sugiyama et al., 2007; King et al., 2010), while other studies found no or positive associations (Kozo et al., 2012; Teychenne, Ball & Salmon, 2012; Chastin et al., 2015). Furthermore, it might be that other factors like the home environment (e.g., number of screens in the home, having a TV in the bedroom; Jones et al., 2010), or automatic processes and habit strength are more strongly related to changes in screen time than the currently included variables (Conroy et al., 2013).

Some moderating effects of educational level and gender were identified; however one should keep in mind that most of these results were only marginally significant: higher levels of modelling from (grand)children only predicted an increase in active transport in high-educated adults, while only in low-educated adults, a more positive perception of retirement (i.e., perceiving retirement as a start for a more active lifestyle) predicted an increase in leisure-time PA. The two other moderating effects were in the unexpected direction (i.e., in men and high-educated adults, higher modelling from friends predicted an increase in car use). Because only four of the forty examined moderating effects were significant, one can presume that generic interventions focusing on men and women, as well as low- and high-educated retired adults have the potential to be effective. Emphasizing specific aspects in low- or high-educated adults, like a focus on modelling from grand(children) in high-educated adults or on obtaining a positive perception of retirement in low-educated adults, could increase the effectiveness of such interventions.

Strengths of this study firstly include its longitudinal design. Second, we focused specifically on adults who are at the start of retirement, which is an important but currently understudied group in health research. Third, a broad range of PA and sedentary behaviors were examined while previous studies mainly focused on either leisure-time PA or TV viewing time, and not on active transportation or car use. However, some limitations should be acknowledged. First, a relatively small sample of recently retired adults participated in the study, limiting the power of our analyses, the generalizability of our findings, and possibly inducing selection bias. Second, only self-reported PA and sedentary behaviors were included. Third, most included predictors primarily focused on PA and not specifically on sedentary behaviors.

Based on the current findings and limitations, we can formulate some suggestions for future research. First, it is recommended to combine the use of questionnaires with objective assessments of sedentary time. Second, potential sedentary-specific factors (e.g., habit strength of sedentary behaviors, home environmental factors) should be included to further unravel the determinants of changes in car use, screen time and other sedentary behaviors.

## Conclusions

In conclusion, this study showed that walkability characteristics (perceived residential density) and self-efficacy at the start of retirement can predict longitudinal changes in active transportation and leisure-time PA. Few moderating effects of gender and educational level were found, so health interventions at the start of retirement, focusing on self-efficacy and specific walkability characteristics, could be effective to increase PA in recently retired adults. No firm conclusions can be drawn on the importance of the examined predictors to explain change in car use and screen time because the explained variances of these regression models were small. It is possible that other factors like the home environment, or automatic processes and habit strength, are more important to explain sedentary behaviors.

##  Supplemental Information

10.7717/peerj.3242/supp-1 Data S1 Longitudinal study—retired adults SPSS data file with raw data used for analyses.

10.7717/peerj.3242/supp-2 Data S2 Longitudinal study—retired adults Data file with raw data used for analyses.

## Additional Information and Declarations

### Data Availability

The following information was supplied regarding data availability: The raw data has been supplied as a Supplementary File.
